# Cardiovascular Symptoms, Dysautonomia, and Quality of Life in Adult and Pediatric Patients with Hypermobile Ehlers-Danlos Syndrome: A Brief Review

**DOI:** 10.2174/011573403X271096231203164216

**Published:** 2024-01-24

**Authors:** Amanda Hertel, William R. Black, Lindsey Malloy Walton, Julie R. Martin, Jordan T. Jones

**Affiliations:** 1 School of Medicine, University of Kansas Medical Center, Kansas City, Kansas, USA;; 2 Department of Pediatrics, University of Kansas School of Medicine, Kansas City, Kansas, USA;; 3 Center for Children's Healthy Lifestyles and Nutrition, Children's Mercy Kansas City, Kansas City, Missouri, USA;; 4 Center for Biobehavioral Health, Abigail Wexner Research Institute at Nationwide Children’s Hospital, The Ohio State University, Columbus, USA;; 5 Department of Pediatrics, Children's Mercy Kansas City, Kansas City, Missouri, USA;; 6 Department of Pediatrics, University of Missouri-Kansas City School of Medicine, Kansas City, Missouri, USA

**Keywords:** Dysautonomia, postural orthostatic tachycardia syndrome, Ehlers-Danlos syndrome, hypermobile ehlers-danlos syndrome, pediatrics, cardiology, quality of life, echocardiogram, composite autonomic symptom scale, cardiovascular symptoms

## Abstract

**Background:**

Hypermobile Ehlers-Danlos Syndrome (hEDS) is a connective tissue disorder characterized by joint hypermobility and other systemic manifestations, such as cardiovascular symptoms, musculoskeletal pain, and joint instability. Cardiovascular symptoms, such as lightheadedness and palpitations, and types of dysautonomia, including postural orthostatic tachycardia syndrome (POTS), are frequently reported in adults with hEDS and have been shown to negatively impact quality of life (QoL).

**Objective:**

This brief review will be an overview of co-occurring symptoms in POTS and hEDS to inform potential cardiovascular screening procedures.

**Results:**

While many patients with hEDS report cardiovascular symptoms, few have structural abnormalities, suggesting that dysautonomia is likely responsible for these symptoms. One validated screening measure for dysautonomia symptom burden is the Composite Autonomic Symptom Scale (COMPASS-31). Studies have found that adults with POTS, hEDS, and both POTS and hEDS have higher COMPASS-31 scores than the general population, suggesting a high symptom burden due to dysautonomia, which leads to impaired QoL.

**Conclusion:**

While studies have examined cardiovascular symptoms and the impact of dysautonomia in adults with and without hEDS, there is scant literature on dysautonomia in pediatric patients with hEDS. Therefore, more studies on cardiovascular symptoms and dysautonomia, as they relate to the quality of life in pediatric patients with hEDS, are needed. This brief review summarizes the current literature on dysautonomia and cardiovascular symptoms in pediatric and adult populations with hEDS.

## 1 INTRODUCTION

Ehlers-Danlos Syndrome (EDS) is a family of connective tissue disorders that include skin hyperextensibility, joint hypermobility, and tissue fragility. The 2017 International Classification of the Ehlers-Danlos Syndromes recognizes 13 subtypes of EDS [1], while a 14^th^ subtype has since been identified [2-4], with the most common subtype being hypermobile Ehlers-Danlos Syndrome (hEDS). Unlike the other EDS subtypes, hEDS has an unknown genetic basis and is diagnosed based on the 2017 International Classification of the Ehlers-Danlos Syndromes [1]. These clinical criteria include generalized joint hypermobility, hyperextensible skin, musculoskeletal pain, joint instability, and other systemic manifestations [1, 5, 6]. In 2023, a new framework was developed specifically for pediatric generalized joint hypermobility and the diagnosis of hEDS in children and young adults [7]. Children innately have higher rates of hypermobility, which may resolve at skeletal and biological maturity, which makes a diagnosis of hEDS using the 2017 classification difficult in pediatric patients [7]. While skeletal maturity continues into adolescence, there is variability around skeletal age compared to chronological age [8, 9], and complete skeletal maturity is variable and for some, may not be obtained until 25 years of age [10]. While there is diagnostic overlap between hEDS and pediatric joint hypermobility, there are differences in the clinical criteria of both these conditions that make them unique, as hEDS has more focus on family history of EDS *versus* pediatric joint hypermobility that has been subdivided into pediatric patients with skin, musculoskeletal, and comorbidities [1, 5].

While hEDS is one of the most common heritable connective tissue disorders, not much is known about the true prevalence of hEDS, and reports predict that all subtypes of EDS combined have a minimum prevalence of 1/5,000. The hEDS subtype accounts for 80 - 90% of all EDS cases [11]. While there is no known genetic etiology for hEDS, it is phenotypically heterogeneous, likely genetically heterogeneous, and is currently thought to have an autosomal dominant inheritance pattern [11, 12]. Additionally, some patients with hypermobile joints have similar symptoms to hEDS but do not meet the hEDS diagnostic criteria and are termed Hypermobility Spectrum Disorders (HSD) [13-15]. Common comorbidities in hEDS include fatigue, abdominal pain, gastroesophageal reflux, headaches, and types of dysautonomia, such as postural orthostatic tachycardia syndrome (POTS) [11, 16-20]. While hEDS is associated with comorbidities and reduced quality of life, it does not have a direct impact on life expectancy [21]. The treatment and management of hEDS in both adults and children includes a multidisciplinary approach [22-25], including physical therapy [26-29], medical devices, such as orthotics [27, 28, 30], exercise focused intervention [31-34], psychological interventions [35-40], and some medications like NSAIDs to help with pain control [28].

The purpose of this brief review is to summarize the current literature on cardiovascular symptoms and dysautonomia as they relate to the quality of life in adult and pediatric patients with hEDS.

## 2 CARDIOVASCULAR SYMPTOMS AND DYSAUTONOMIA

In a recent study on adults and children with hEDS, most patients with hEDS (89%) report at least one cardiovascular symptom, with the most common cardiovascular symptoms reported shown in Fig. (**1**) [41]. In the study, 30% of those with hEDS also reported a diagnosis of POTS [41]. Interestingly, the majority (83%), but not all of the patients with hEDS had an echocardiogram, with over 90% showing trivial to mild valvular insufficiency, while 8% had mitral valve prolapse (MVP), and none had aortic root dilation (ARD) [41]. These valvular insufficiency, MVP, and ARD percentages are similar to reports in the general population without EDS [41]. Some studies have reported similar cardiovascular findings in patients with hEDS [42-44], while others report much higher ARD percentages ranging from 14 to 17% [45, 46]. These discrepancies are likely due to variations within the hEDS population but may also be related to the variation in sample size and patient demographics [41]. Selection bias is another important consideration, as some studies recruit from EDS-specific clinics while others look at the general population. Another report suggested that echocardiography may not be indicated for patients with hEDS but might be indicated in cases with positive family history of cardiovascular conditions, abnormal physical exams, or patients with hEDS who have primarily cardiovascular symptoms [47]. These findings show that many patients with EDS report cardiovascular symptoms; however, few have significant cardiac anatomic abnormalities noted on echocardiogram or electrocardiogram [41]. Those who did have cardiac anatomic abnormalities had MVP or trivial prolapse as shown on echocardiogram and only one patient had a clinically significant electrocardiogram finding of supraventricular tachycardia (SVT) [41]. The discrepancy between symptoms and anatomic abnormalities suggests that dysautonomia is the primary cause of the cardiovascular symptoms experienced by patients with hEDS.

In the general population, POTS has a prevalence of 0.2% [48], but the prevalence of dysautonomia in patients with hEDS ranges from 31 to 94% [20]. In adults, POTS is diagnosed when all the following criteria are met: An increased heart rate of 30 or more beats per minute when transitioning from supine to standing, no orthostatic hypotension present, and chronic symptoms associated with standing. Given that adolescents have a higher resting heart rate than adults, the diagnostic cut-off for minimum heart-rate elevation for adolescents (12 - 19 years) increases to 40 or more beats per minute [48]. Other common symptoms of POTS in both adolescents and adults include syncope, pre-syncope, lightheadedness, and other autonomic symptoms like diarrhea, fatigue, or migraines [30, 48-50]. The Heart Rhythm Society and collaborators recommend that a complete history, physical, orthostatic vitals, and ECG be performed at the time of POTS diagnosis to ensure other conditions are ruled out [48]. While POTS is a chronic condition, it is not associated with early mortality, and proper treatment can help resolve symptoms over time [51]. Some common treatment modalities for POTS in adults include non-pharmacologic therapies like increased salt and fluid intake [52, 53], exercise training [52, 53], and compression garments [54], as well as pharmacologic options, such as midodrine (vasoconstrictor) [53, 54], beta-blockers [53, 54], and fludrocortisone (volume expander) [53, 54]. The non-pharmacologic treatments are the same for pediatric patients with POTS and are the preferred treatment, with pharmacologic options only being used off-label in cases where non-pharmacologic modalities cannot adequately control symptoms [55, 56]. There are currently no treatment guidelines specific for pediatric patients with both POTS and hEDS. Half of all patients with POTS are diagnosed during adolescence [57]; however, while POTS in adult patients has been well-documented [48, 57-61], there is scant literature on POTS in pediatric patients [62].

## 3 STUDIES ON POTS AND HEDS

There is a strong correlation between POTS and hEDS, and it has been suggested that if an individual meets the criterion for POTS, they should also be screened for hEDS [58, 63]. One study evaluated patients with POTS and found that 31% met the criteria for a diagnosis of hEDS [58]. Other studies have shown that 16% [59] and 22% [64] of adult and adolescent patients, respectively, with a POTS diagnosis also met hEDS diagnostic criteria. Additionally, in both adult and pediatric patients diagnosed with POTS, more than 50% also meet the criteria for either hEDS or joint hypermobility syndrome (JHS, the previously used term for HSD) [58, 59, 64].

Significant symptom burden and a high level of impairment are associated with dysautonomia in patients with hEDS [65]. Part of the impairment may be attributed to delays in diagnosis (an average of 2 years) and the need to see numerous physicians before a dysautonomia diagnosis, postponing intervention efforts [57]. This is concerning as most patients report an onset of symptoms between 14 to 17 years of age, which may significantly impact social engagement with peers, social development [57], and the development of self-identity [66]. These studies demonstrate the importance of recognizing and accurately diagnosing POTS and other forms of dysautonomia as early as symptoms develop [57], particularly in patient populations prone to these types of symptoms, such as those with hEDS [65].

One of the earliest studies to describe the relationship between JHS and dysautonomia was conducted in 2004. This case-control study included 170 adult females diagnosed with JHS and matched controls who completed symptom surveys [61]. Of the participants, 41% of patients with JHS had at least one pre-syncopal symptom compared to 15% in the control group. Additionally, pain and fatigue were the most common symptoms reported in the JHS group (91% and 71%, respectively) [61]. Another study on 48 adult patients with JHS and 30 controls without JHS found that all those with JHS reported at least five orthostatic symptoms lasting six or more months. In contrast, only 3 out of 30 control participants reported five or more orthostatic symptoms. Further evaluation revealed that 22% of those with JHS also met orthostatic hypotension criteria, while 34% met POTS criteria, and the remainder of the participants with JHS were categorized with orthostatic intolerance [67]. These studies demonstrate that orthostatic symptoms, pain, and fatigue are common in patients with hypermobility. However, these findings are limited to adults with hypermobility, and the prevalence and natural history of cardiovascular manifestations in pediatric hEDS are poorly characterized [68].

Many adult-focused studies reported that patients with POTS report numerous clinical impairments in addition to their dysautonomia (*e.g*., sleep disturbance, fatigue, and anxiety) [60] and that symptoms improve over time with a continuation of treatment [69], including some with total symptom resolution [70]. However, it is unclear if these results may be generalized to a pediatric population due to a lack of literature on dysautonomia and hEDS in pediatric patients [68].

Few studies have reported dysautonomia in pediatric patients with hEDS despite half of all patients with POTS being diagnosed during adolescence [62]. Some studies have examined the clinical overlap between chronic fatigue syndrome (CFS) and hEDS in the setting of POTS symptoms. CFS is diagnosed by otherwise unexplained profound fatigue that is not improved after rest and an inability to perform pre-illness activities for at least six months in addition to other symptoms [71]. Some common symptoms of CFS include muscle and joint pain, fatigue, and orthostatic intolerance [72]. One study found that in pediatric patients with hEDS and CFS, 83% also met the criteria for POTS [63]. This compelled the authors to recommend that patients diagnosed with CFS or dysautonomia should be screened for EDS as common clinical practice [63]. In a study on 362 pediatric patients with POTS, 20% met the 2017 International classification criteria for EDS, and 33% were diagnosed with HSD. This further illuminates the link between hEDS, HSD, and POTS and the importance of recognizing comorbidities in these conditions [73]. Further studies need to be done to better understand the relationship between hEDS and dysautonomia in those with EDS, particularly in the pediatric population.

## 4 COMPASS-31, AUTONOMIC SYMPTOM BURDEN, AND QUALITY OF LIFE

The availability and accessibility of well-researched screening measures are paramount to better understanding the relationship between hEDS and dysautonomia. One validated and well-established Patient Reported Outcome (PRO) that assesses autonomic dysfunction is the COMPASS-31 (Composite Autonomic Symptom Scale). This survey was developed by the Mayo Clinic based on a statistical analysis of the 169-question Autonomic Symptom Profile (ASP) and its scoring instrument, the Composite Autonomic Symptom Score (COMPASS). Researchers simplified the original ASP into 31 questions under six domains, which is now known as the COMPASS-31. The six domains measured include orthostatic intolerance, vasomotor, secretomotor, gastrointestinal, bladder, and pupillomotor. Higher scores for the COMPASS-31 indicate increased dysautonomia symptoms, making it a commonly used PRO in studies evaluating dysautonomia [74].

The COMPASS-31 was used to assess autonomic symptoms in patients with POTS compared to healthy controls in an adult cohort. Those with POTS had significantly higher COMPASS-31 sub-domain and total scores. The sub-domains with the greatest difference between the two groups were orthostatic intolerance and pupillomotor domains. This study also showed that patients with POTS have similar total COMPASS-31 scores to patients with neuropathy, supporting the idea that patients with POTS have a significant autonomic symptom burden [75]. Other studies have also found that the orthostatic intolerance sub-domain had the most significant contribution to total COMPASS-31 scores at baseline in patients with POTS [69, 70]. These studies suggest that orthostatic intolerance and pupillomotor sub-domains and related symptoms should be more closely monitored in patients with POTS during clinical assessments. Knowing the importance of these sub-domains could also help guide decision-making in diagnostic evaluation, symptom management, and treatment [69, 70, 75].

Longitudinal studies on POTS found a statistically significant and clinically meaningful improvement in total COMPASS-31 scores at one-year follow-up appointments [69, 70]. The treatment modalities varied, ranging from non-pharmacologic treatments like increased salt and water intake and progressive physical activity to pharmacologic treatments like beta blockers, midodrine, and fludrocortisone. This suggests that dysautonomia symptoms in patients with POTS can improve over time with treatment, regardless of the treatment modality, based on the decreased COMPASS-31 scores at follow-up [69, 70].

Several studies have also examined different aspects of quality of life (QoL) as a measure of well-being and function in patients with dysautonomia and POTS. It was found that health-related QoL (HRQoL), as measured with the 36-Item Short Form Health Survey (SF-36), improved over time in most adolescents and young adults with POTS. It was found that between 2 to 10 years after a POTS diagnosis, most patients (51%) had improved symptoms, and 19% reported complete resolution of symptoms when continuing treatment (regardless of treatment modality). Therefore, in addition to improved symptom burden, the level of impairment and impact on QoL caused by POTS may also improve over time with appropriate treatment [76].

While it has been shown that POTS has a significant impact on QoL, additional studies have shown that the QoL for patients with hEDS and those with both hEDS and POTS are also dramatically impacted. In a study utilizing the Autonomic Symptom Profile (ASP) and SF-36, the autonomic symptom burden of hEDS was similar to patients with fibromyalgia and significantly higher than healthy controls and people with other subtypes of EDS. Those with hEDS had lower QoL, higher fatigue, and higher pain severity with increasing autonomic symptom burden [77]. In another study comparing patients with POTS to those with POTS and hEDS, the median total COMPASS-31 and SF-36 scores were not statistically different across both groups. The impact of autonomic symptoms on QoL is similar for patients with POTS to those with POTS and hEDS. While both conditions have been shown to significantly impact autonomic function and QoL, the relationship between POTS, dysautonomia, and hEDS must be further explored [58] to better understand their comorbidity and explore potentially shared causal mechanisms.

## SUMMARY AND CONCLUSION

Many adults with hEDS suffer from dysautonomia, and a significant portion of them experience cardiovascular symptoms as part of their dysautonomia. However, few adults with hEDS have structural cardiovascular abnormalities, suggesting that dysautonomia is primarily responsible for cardiovascular symptoms. Additionally, dysautonomia has a significant impact on QoL for adults with hEDS. This suggests that if generalized to pediatric hEDS, dysautonomia should receive greater consideration for early evaluation and treatment, with less emphasis on cardiovascular anatomical abnormalities. It may be that diagnostic and intervention efforts should first focus on dysautonomia over structural cardiovascular disease, which is not as prevalent. While numerous studies have assessed cardiovascular symptoms, dysautonomia-related symptoms, and functioning in adults with and without EDS, this work has been mostly limited to the adult population. Such work may not be generalized to pediatric patients. Studies focusing on cardiovascular symptoms and dysautonomia as they relate to the quality of life in pediatric patients with hEDS are needed to determine if similar impacts are present. Additionally, no current studies evaluate COMPASS-31 scores and QoL measures in pediatric populations with hEDS, and symptom onset and impact of symptoms may differ from adult populations with hEDS. Furthermore, early identification and awareness could lead to earlier diagnosis, treatment, and better QoL. Future studies that focus solely on pediatric patients with hEDS are needed to determine similarities and differences compared to adults with hEDS.

## Figures and Tables

**Fig. (1):**
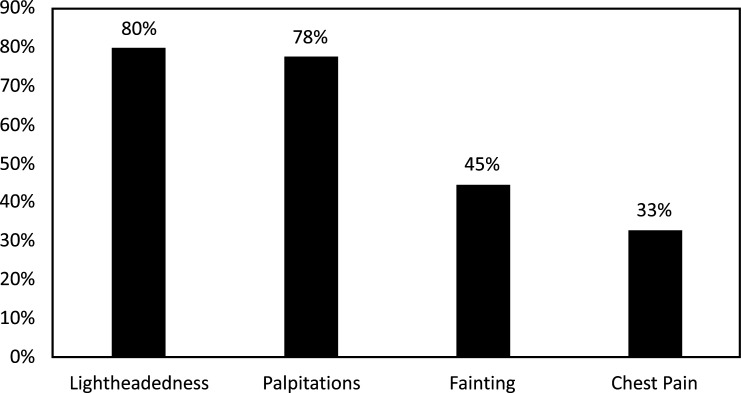
Frequency of cardiovascular symptoms in patients with hEDS.

## LIST OF ABBREVIATIONS

hEDS: Hypermobile Ehlers-Danlos Syndrome
POTS: Postural Orthostatic Tachycardia Syndrome
QoL: Quality of Life
HSD: Hypermobility Spectrum Disorder
PRO: Patient Reported Outcome
COMPASS: Composite Autonomic Symptom Scale
MVP: Mitral Valve Prolapse
ARD: Aortic Root Dilation
CFS: Chronic Fatigue Syndrome
JHS: Joint Hypermobility Syndrome
SF-36: 36-Item Short Form Health Survey
